# Experimental Investigation of the Machining Characteristics in Graphite-Powder-Mixed Electrochemical Discharge Machining of Microholes in Glass

**DOI:** 10.3390/mi14101810

**Published:** 2023-09-22

**Authors:** Weidong Tang, Jikai Yao, Jize Zhang, Quancai Zhao, Lixiang Fan, Cong Mao, Xiaoming Kang, Xuyu Li, Shuhan Chen

**Affiliations:** 1 College of Automotive and Mechanical Engineering, Changsha University of Science and Technology, Changsha 410114, China; wdtang@csust.edu.cn (W.T.);; 2 State Key Laboratory of Mechanical System and Vibration, School of Mechanical Engineering, Shanghai Jiao Tong University, Shanghai 200240, China

**Keywords:** electrochemical discharge machining (ECDM), microhole, glass, graphite powder

## Abstract

The effect of graphite powder on the machining characteristics in graphite-powder-mixed electrochemical discharge machining of microholes was still not clear. How the discharge mechanism changed with the addition of graphite powder into the electrolyte, which further led to changes in the morphology of the machined holes, remained to be revealed. In this study, a series of microhole machining experiments were conducted in glass. Comparisons of the discharge energy, microhole entrance diameter, hole taper, and tool electrode morphology after machining were made when machining in the electrolytes with and without graphite powder. Experimental results revealed that there were a lot of small pulse currents distributed on the current waveform when machining with the graphite-powder-mixed electrolyte. The average discharge energy of the small pulse current was 2.8 times as much as that of the general electrochemical discharge. After introducing graphite powder into the electrolyte, the entrance diameter of the hole became larger when the hole depth was deeper than 200 μm. The HAZ width increased with increasing hole depth at the voltage of 37–41 V, while it decreased at the voltage of 43 V. A reduction in hole taper angle with a range of 0.5° to 2.3° was achieved. In addition, after machining in electrolytes with and without graphite powder, the tool electrode surfaces showed different morphologies due to different discharges.

## 1. Introduction

Electrochemical discharge machining (ECDM) is a promising technology to process insulating hard and brittle materials such as glass, quartz and ceramics [1]. ECDM can be applied to process microholes, microchannels and microstructures in nonconductive materials, which has application in the micro-electro-mechanical system (MEMS), such as micro-accelerometers, micro-reactors, micro-pumps and drug delivery devices.

The workpiece material is removed when the gas film is broken through and electrochemical discharge occurs between the electrode and the electrolyte. The ECDM process is influenced by a number of factors such as electrolyte, gas film, tool electrode and so on [2,3,4]. In order to investigate the mechanisms of discharge and material removal in the ECDM process, considerable research had been conducted. It was found that the gas film around the tool electrode played an important role in the ECDM process. The thickness of the gas film around a cylindrical tool electrode with a diameter of 1 mm had been estimated to be about 50–100 μm [5]. Jiang et al. [6] experimentally measured the thickness of the gas film to be about 24 μm around the tool electrode with a diameter of 0.5 mm. The average formation time of the gas film is generally about 20 μs [7]. Allagui et al. [8] measured the life cycle of the gas film to be about 2–3 ms on a 0.5 mm diameter electrode in a 30% concentration sodium hydroxide solution. When the voltage applied to the gas film was large enough, the electric field in the gas film reached the breakdown threshold. Then, the gas film was broken down and an electrochemical discharge occurred. Basak and Ghosh [9] suggested that there were narrow conduction bridges within the dense gas film around the tool electrode, and the large densities of current passing through these bridges produce transient ion boiling, leading to an electrochemical discharge. Jain et al. [10] proposed that each bubble was a valve, which collapsed under a high electric field to produce a discharge. Behroozfar et al. [11] inferred that the discharge arc column was cylindrical according to the current signal and the circular crater on the workpiece surface. Kulkarni et al. [12] found that the discharge activity was a discrete phenomenon based on the analysis of time-varying current signals.

Many effects have been made to improve the machining quality. Yang et al. [13] proposed a spherical electrode with a ball at the electrode bottom. The ball diameter was larger than the electrode diameter, which avoided the accumulation of gas bubbles at the entrance during the machining, thus improving the surface quality and machining accuracy. Han et al. [14] proposed a sidewall-insulated electrode to achieve partial insulation on the tool electrode sidewall, which improved machining accuracy due to the suppression of discharge at the liquid surface. Tang et al. [15] proposed a diamond-coated side-insulated electrode and achieved microholes with small entrance diameters and good surface integrity. Xu et al. [16] obtained a microhole with 1000 μm machining depth and good surface quality within 100 s by applying a suitable magnetic field to induce magnetohydrodynamic effects which improved the refreshing of electrolyte. Singh et al. [17] integrated ultrasonic vibration into the ECDM of the microhole process to increase the aspect ratio by a factor of five and to reduce energy consumption by a factor of 2.23.

The addition of suitable substances to the electrolyte could also affect the machining results. Jain et al. [18] added artificial bubbles to the electrolyte and obtained a reduction in both overcutting and material removal rate. Wüthrich et al. [19] introduced a soap solution into the electrolyte to reduce the thickness of the gas film and finally improved the machining repeatability. Lin et al. [20] added the ethanol to the electrolyte and applied a magnetic field to explore the machining accuracy on glass. Experimental results showed that the electrolyte flow and bubbles around the electrode were relatively stable under the magnetohydrodynamic effect, and the roundness of the machined hole was significantly improved and the hole taper improved by 30%. Yang et al. [21] introduced suspended particles of silicon carbide into the electrolyte for wire electrochemical discharge machining (WECDM) and found that the surface quality of the machined workpiece was effectively improved using this method. The reason was that the grinding action of the free particles in the flowing fluid at the machining zone resulted in the removal of thermal cracks and heat-affected layers, so the surface quality of the machined surface was effectively improved. Elhami et al. [22] added nanoparticles to the electrolyte and the experimental results showed that the nanofluid enhanced both the electrical and thermal conductivities, resulting in stronger discharge and higher material removal rate. The results demonstrated that the hole depth was enhanced by 21.1% and 18.7% for Cu and Al_2_O_3_ nanoelectrolytes, respectively.

Graphite powder has attracted a lot of attention in the field of EDM due to its excellent electrical and thermal conductivity. Unses et al. [23] observed a significant improvement in EDM of Ti-6Al-4V when adding graphite powder to kerosene. Jahan et al. [24] investigated the effect of kerosene mixed with graphite powder (Gr), aluminum (Al) and alumina (Al_2_O_3_) nanopowders, respectively, on the EDM of tungsten carbide. The results showed that the powder particle size, concentration, density, electrical conductivity and thermal conductivity were the most important factors affecting the powder performance. Kim et al. [25] found that in the EDM with graphite powder hybrid kerosene, the machining time and tool wear length were reduced by 30.9% and 28.3%, respectively, compared to those in the EDM with conventional kerosene. Reddy et al. [26] investigated the EDM process of stainless steel PH17-4 using a mixed dielectric with both graphite powder and surfactant, and a uniform discharge distribution and an improved surface integrity were achieved.

In recent years, graphite powder had also been applied in ECDM. Han et al. [27] added graphite powder to the electrolyte to improve surface integrity. Varghese et al. [28,29] studied the optimal parameters in graphite-powder-mixed ECDM. It was shown that the maximum material removal rate was achieved in the machining of borosilicate glass at a voltage of 40 V, graphite powder concentration of 1.25%, duty cycle of 70% and tool rotation speed of 500 rpm. They also studied the influence of tool electrode diameter on the electrochemical discharge machining with graphite-powder-mixed electrolyte and found that a smaller tool electrode diameter resulted in a higher material removal rate and lower overcut [30].

The current research on graphite-powder-mixed electrochemical discharge machining (GPM-ECDM) is mostly focused on machining performance. It remains to be revealed how the discharge mechanism changes when graphite powder is added to the electrolyte, which further leads to changes in the morphology of the machined holes. Therefore, in this study, the discharge mechanisms in GPM-ECDM were studied, and the influence of graphite powder on the hole entrance diameter, heat-affected zone (HAZ), hole taper and tool electrode morphology were investigated systematically.

## 2. Materials and Methods

### 2.1. Experimental Setup

**Figure 1:**
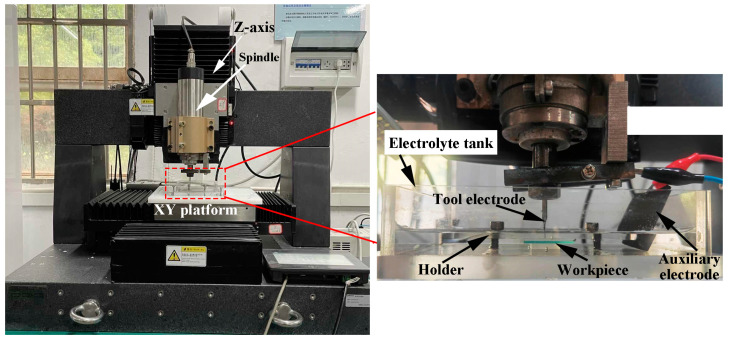
Diagram of the experimental setup.

The experimental setup is shown in Figure 1. It consisted of a precision three-axis machine tool and an ECDM unit. The Z-axis of the machine was equipped with a precision electric spindle with a rotational error within 2 μm. The ECDM unit mainly consisted of an electrolyte tank, a workpiece, a tool electrode, an auxiliary electrode, a pulsed DC power supply, a current probe and an oscilloscope. The tool electrode was fixed to the electrical spindle, and the electrolyte tank was mounted on the XY platform. An oscilloscope (RIGOL TECHNOLOGIES Co., Ltd., Beijing, China. Model: RIGOL MSO5000) was used to collect the voltage and current signals during the machining.

### 2.2. Experimental Procedure

A soda-lime glass with a size of 5.4 mm × 76.2 mm × 1 mm was used as the workpiece, which was immersed in a 6 mol/L sodium hydroxide solution with an electrolyte level of 2 mm. The auxiliary electrode was made of graphite with a dimension of 60 mm × 50 mm × 1.5 mm. The graphite powder used in the experiments was artificial graphite (content ≥99.5 wt %) with an average particle size of 1 μm. The material properties of graphite powder are shown in Table 1.

**Figure 2:**
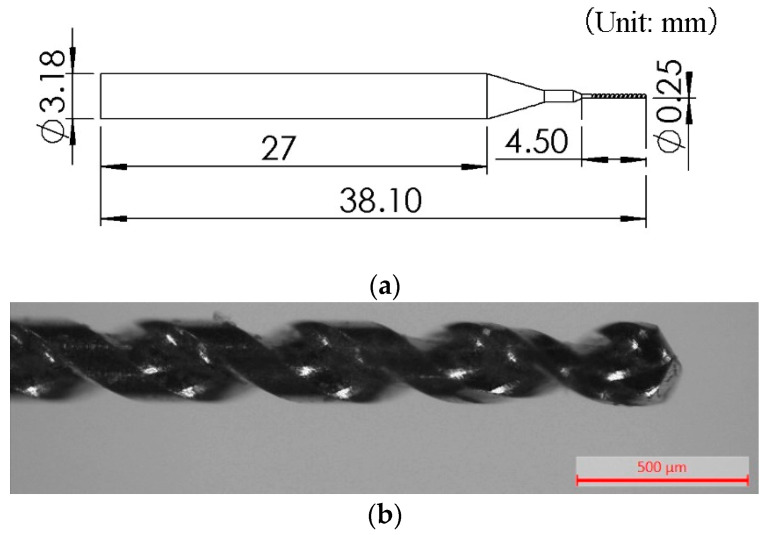
The dimension and shape of the microdrill bit. (**a**) The dimension of the microdrill bit. (**b**) The working part of the microdrill bit.

A cemented carbide microdrill bit was used in the experiments. The diameter of the working part was 0.25 mm. Figure 2 shows the dimension and shape of the microdrill bit.

In the ECDM experiments, a pulsed power supply with a frequency of 500 Hz and a duty cycle of 50% was applied. Four sets of voltages from 37 to 43 V (with an interval of 2 V) were used in the experiments. The voltage and current waveforms were collected from the ECDM experiments with graphite-powder-mixed electrolyte or the conventional electrolyte. Comparisons of the collected compared voltage and current waveforms were conducted, and the difference in the current waveforms caused by introducing graphite powder to electrolyte was explained.

Microhole machining experiments by ECDM using electrolyte with and without graphite powder were conducted with a constant-feed method. The hole entrance diameter and heat-affected zone (HAZ) at the entrance were measured and compared for different machining depths at different voltages. Microholes with a depth of 100–400 μm were machined in electrolytes with and without graphite powder. The hole tapers were calculated and compared. In addition, the tool electrode morphologies after machining in different electrolytes were analyzed. The experiments were repeated three times under each set of machining parameters during the experiments, and the experimental results such as the hole entrance diameter, HAZ and hole taper were the average value of the three experiments. The machining parameters are shown in Table 2.

## 3. Results and Discussion

### 3.1. Influence of Graphite Powder on Current Signal

**Figure 3:**
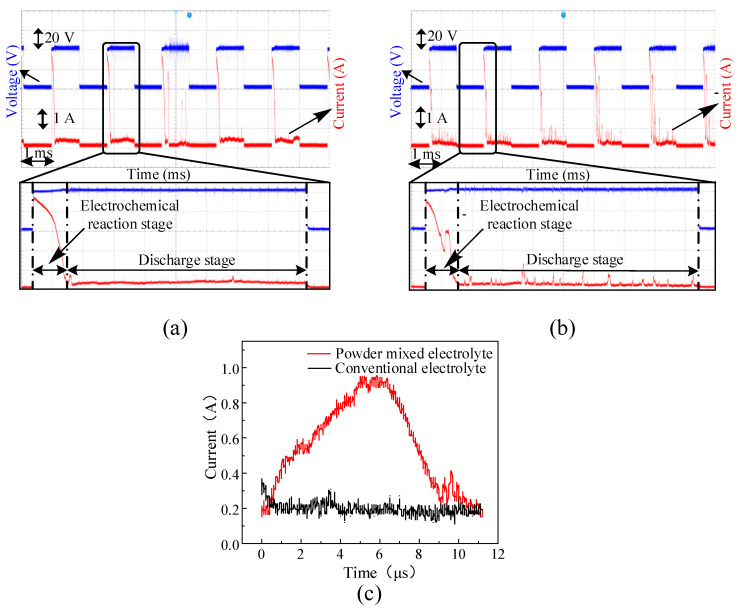
The voltage and current signals in different electrolytes at the voltage of 41 V. (**a**) The conventional electrolyte. (**b**) The graphite-powder-mixed electrolyte. (**c**) Comparison between the small pulse current waveform and the conventional electrochemical discharge current waveform.

Figure 3 shows the voltage and current signals collected from the hole machining process with two different electrolytes at the voltage of 41 V. Figure 3a,b show the voltage and current waveforms collected from the conventional electrolyte and graphite-powder-mixed electrolyte, respectively. The pulse period was 2 ms. A period of the current signal can be divided into two stages [31]. The former part of the current signal is the electrochemical reaction stage. At this stage, a lot of bubbles are produced to form a complete gas film around the tool electrode through the electrolysis reaction. The latter part of the current signal is the discharge stage where the gas film is broken down and an electrochemical discharge is generated.

It can be seen from Figure 3a,b that, at the discharge stage, there were significant differences in the discharge currents collected from the conventional electrolyte and the graphite-powder-mixed electrolyte, respectively. Compared to the general discharge current in the conventional electrolyte, the discharge current collected from the graphite-powder-mixed electrolyte showed a lot of additional small pulse currents. Figure 3c illustrates a comparison between the small pulse current and the conventional discharge current. As shown in the figure, the pulse width of the small pulse current was 11 μs, with 6 μs at the rising edge and 5 μs at the falling edge. The peak value of the small pulse current was 0.96 A, while the conventional discharge current was more stable and the discharge current value was in the range of 0.15–0.3 A.

In the graphite-powder-mixed electrolyte, the small pulse currents were not continuous but occurred discretely in the current waveforms at the discharge stage. This phenomenon can be clearly observed in Figure 3b. The occurrence of the small pulse current was attributed to the addition of graphite powder to the electrolyte. The graphite particle in the electrolyte was influenced by Brownian motion, van der Waals forces, buoyancy, gravity and so on. Therefore, the graphite particle exhibited irregular and random motion in the electrolyte. A large number of graphite particles were randomly distributed in the electrolyte, and some graphite particles were located at the gas–liquid interface between the gas film and the electrolyte. Generally speaking, the electrochemical discharge occurs between the tool electrode and the electrolyte. When a graphite particle located on the gas–liquid interface was close enough to the tool electrode, the gas film was broken down, and a discharge occurred between the tool electrode and the graphite particle. Therefore, the small pulse current appeared in the current waveform at the discharge stage. The graphite particle was then broken or crushed into the electrolyte by the discharge. Thereafter, when the distance between the tool electrode and the electrolyte was short enough, the gas film was broken down, and the discharge switched to the general electrochemical discharge between the tool electrode and the electrolyte.

The discharge energy during the discharge stage can be calculated using Equation (1), where *Q* represents the discharge energy, *U* and *I* are the applied voltage and average current during the discharge pulse width, respectively, and *t* is the discharge pulse width time.

(1) Q=U×I×t

In Figure 3c, the average current of the small pulse current during an 11 μs pulse width is 0.56 A. Substituting the values into Equation (1), the energy of the small pulse discharge between the tool electrode and graphite was calculated as *Q* =$\;2.53\times 10^{-4}$ J, while during the same period, the average current in the conventional electrolyte was 0.2 A. The energy of the conventional electrochemical discharge between the tool electrode and electrolyte was *Q* = ${0.9\times 10}^{-4}$ J. Therefore, it can be seen that the discharge energy of the small pulse current in the graphite-powder-mixed electrolyte was 2.8 times as much as that in the conventional electrolyte for the same discharge duration. Throughout the entire discharge stage, since multiple small pulse discharges occurred in the graphite-powder-mixed electrolyte, the average discharge energy was larger than that in the conventional electrolyte.

### 3.2. Entrance Diameter of the Microhole

During the ECDM of the microhole process, a significant amount of sidewall discharge and thermal-assisted chemical etching occurred at the entrance of the hole due to the accumulation of discharge heat and sufficient electrolyte supply at the hole entrance, resulting in the formation of a tapered entrance edge.

**Figure 4:**
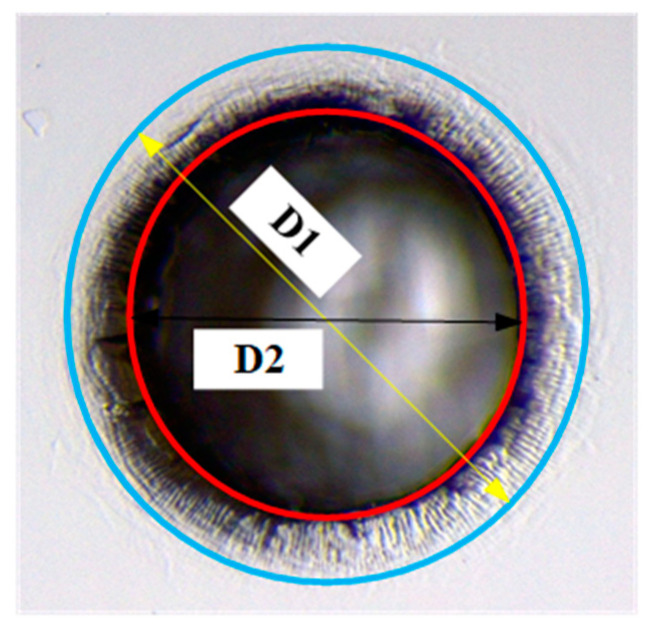
Schematic of the microhole entrance.

Figure 4 shows the schematic of the microhole entrance machined by ECDM. The material removal area of the microhole entrance was divided into two regions by two concentric circles. The large circle with a diameter of D1 covered all material removal areas at the hole entrance. The small circle with a diameter of D2 referred to the area where a significant amount of material removal occurred and has a certain depth. In this study, the diameter of the large circle (D1) was defined as the large entrance diameter, and the diameter of the small circle (D2) was defined as the small entrance diameter. The difference between the two areas was defined as the heat-affected zone (HAZ), and (D1 − D2)/2 defines the width of the HAZ.

**Figure 5:**
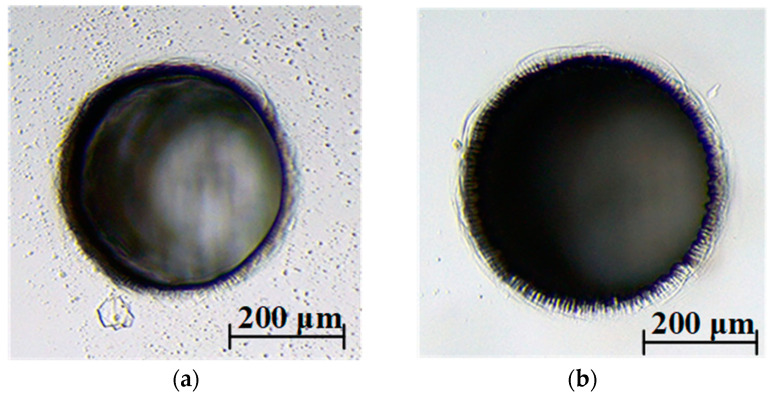
Microholes with a depth of 400 μm machined in different electrolytes. (**a**) Conventional electrolyte. (**b**) Graphite-powder-mixed electrolyte.

Figure 5 shows the morphologies of microholes with a depth of 400 μm in soda-lime glass machined with different electrolytes. The machining voltage was 37 V. Compared to the appearance of the hole entrance obtained in the conventional electrolyte, the addition of graphite powder into the electrolyte resulted in a more intense etching effect at the hole entrance. Measurements of the microholes showed that the large entrance diameters were 345 μm and 390 μm, and the small entrance diameters were 305 μm and 339 μm for the conventional electrolyte and the graphite-powder-mixed electrolyte, respectively. The experimental results indicated that the diameter of the holes was increased and the chemical etching zone at the hole entrance was more significant in graphite-powder-mixed electrolyte.

**Figure 6:**
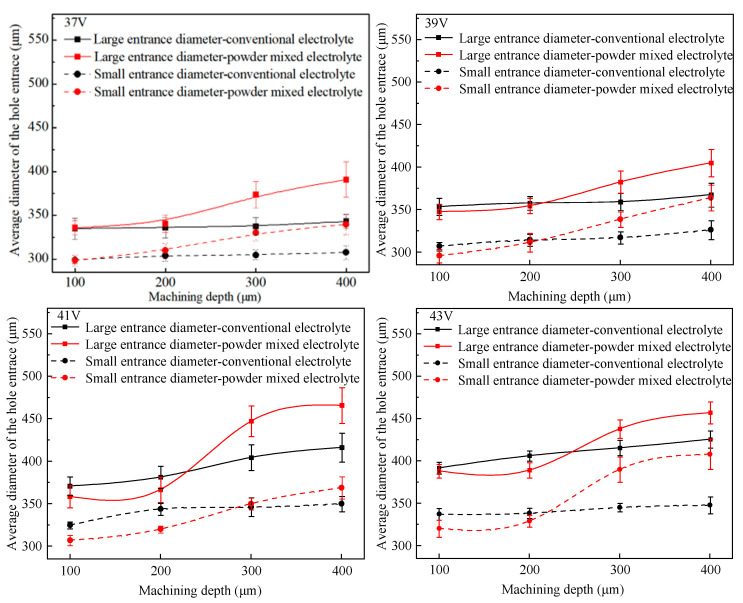
Variation of the average diameter of the hole entrance with machining depth.

In order to compare the variation of hole entrance diameters with hole depth increasing, several sets of microhole machining experiments were carried out by varying the machining voltage. Detailed machining parameters are displayed in Table 2. The average diameters of the large and small entrances of the holes obtained with different electrolytes were calculated, and the results are shown in Figure 6.

Figure 6 shows that when the machining depth was lower than 250 μm, there was little difference in the large entrance diameter for the conventional electrolyte and the graphite-powder-mixed electrolyte. The large entrance diameter of microholes machined in conventional electrolyte nearly remained stable as the hole depth increased at the voltages of 37 V and 39 V. At the voltages of 41 V and 43 V, it increased slowly with hole depth. When using graphite-powder-mixed electrolyte, the large entrance diameter of microholes increased slowly with increasing hole depth when the depth was lower than 250 μm, and it was almost equal to that obtained in the conventional electrolyte, with a difference in the range of 3–17 μm. However, when the depth was deeper than 250 μm, the large entrance diameter of microholes machined using graphite-powder-mixed electrolytes increased relatively rapidly with increasing hole depth, and it was larger than that machined in conventional electrolyte.

The difference in hole diameter variations at different depths was related to the different electrolyte supply in the machining zone. When the hole depth was shallow and the electrolyte supply was sufficient, the discharge at the electrode bottom was more intense, resulting in more material removal at the electrode bottom and a larger gap at the bottom. Therefore, the electrolyte was more likely to flow into the bottom of the hole, and less gas film accumulated at the hole entrance. Thus, the entrance diameter of the microhole obtained in graphite-powder-mixed electrolyte was almost equal to that of the microhole obtained in the conventional electrolyte. However, when the depth exceeded 250 μm, the electrolyte had difficulty entering the machining zone at the bottom of the hole. Therefore, the discharges were concentrated at the hole entrance. Due to the higher discharge energy in the graphite-powder-mixed electrolyte, the hole entrance diameter increased rapidly.

In the conventional electrolyte, when the machining depth increased from 100 μm to 400 μm, the small entrance diameter increased slowly. The increased rates were 2.6%, 6.3%, 7.8% and 3.1% for the machining voltages of 37 V, 39 V, 41 V and 43 V, respectively. However, for microholes machined using graphite-powder-mixed electrolyte, the increase in small entrance diameter was greater than that of microholes machined in conventional electrolyte under the same machining parameters. The increased rates were 13.8%, 23.1%, 20.2% and 27.4% for the machining voltages of 37 V, 39 V, 41 V and 43 V, respectively. When the machining depth was in the range of 100–200 μm, the small entrance diameter of the holes machined in the graphite-powder-mixed electrolyte was equal to or slightly less than that of holes obtained in conventional electrolyte. This was because, for a hole depth less than 200 μm, the electrolyte supply at the electrode bottom was sufficient, and the discharge energy in the graphite-powder-mixed electrolyte was relatively high. Therefore, a large amount of material was removed below the electrode bottom, resulting in a larger gap between the electrode bottom and the hole bottom. This gap facilitates the electrolyte entering the hole bottom, resulting in more discharges at the hole bottom. Therefore, the discharge on the electrode sidewall was relatively reduced, resulting in smaller diameters of the small entrance of the holes.

In the range of 200–300 μm, the small entrance diameter increased rapidly and exceeded that of the hole obtained in conventional electrolyte. This was due to the fact that when the hole depth exceeded 250 μm, it was difficult for the electrolyte to flow into the hole bottom. Therefore, the discharge at the hole bottom decreased. In contrast, the discharge at the sidewall of the tool electrode increased accordingly, thus increasing the small entrance diameter of the hole.

### 3.3. Heat-Affected Zone

**Figure 7:**
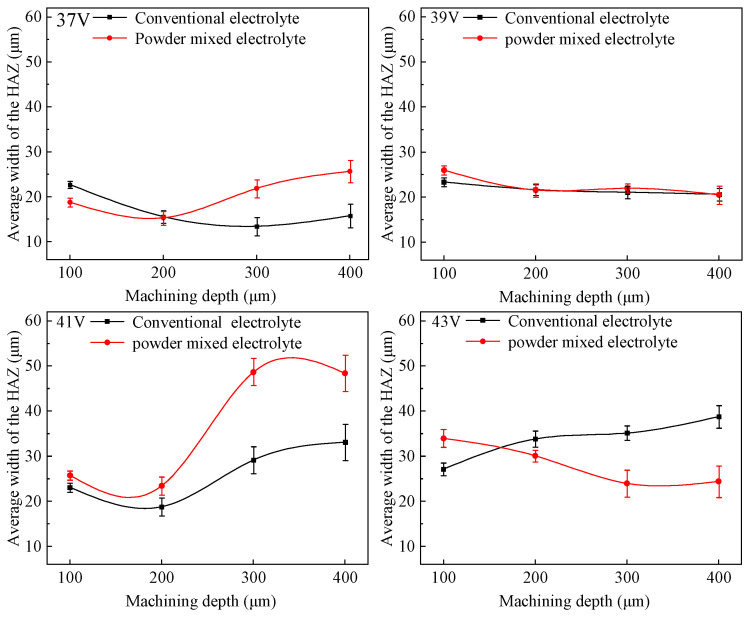
Variation of HAZ width with machining depth.

The heat-affected zone (HAZ) is an important indicator for evaluating the machining quality of microholes machined by ECDM [15]. In the field of ECDM of non-conductive material, the rounded corner area at the hole entrance edge was considered as the heat-affected zone, which was the annular region between the large circle and small circle in Figure 4. Figure 7 shows the width of the HAZ of the microholes machined with two different electrolytes at different voltages.

The HAZ width was influenced by both the large and small entrance diameters. An increase in the HAZ width indicated that the increase in the large entrance diameter was greater than the increase in the small entrance diameter, while a decrease in the HAZ width indicated that the increase in the large entrance diameter was less than the increase in the small entrance diameter.

It can be seen from Figure 7 that at 37 V and 39 V, the HAZ width of the machined hole does not vary significantly with increasing machining depth from 100 μm to 400 μm in both the conventional and graphite-powder-mixed electrolytes, indicating that the increasements in both the large and small entrance diameters were approximately equal.

At the voltage of 41 V, the HAZ width in both electrolytes exhibited an increasing trend as the machining depth increased from 100 μm to 400 μm. A comparison of the two curves showed that the HAZ width of the hole machined in the graphite-powder-mixed electrolyte was larger than that in the conventional electrolyte. This was because, in both electrolytes, the increased rate of the large entrance diameter was higher than that of the small entrance diameter. In particular, the increased rate of the large entrance diameter in the graphite-powder-mixed electrolyte was faster than that in the conventional electrolyte, thus resulting in a larger HAZ width.

Under the voltage of 43 V, the HAZ width of the hole machined in the conventional electrolyte continued to increase as the machining depth increased from 100 μm to 400 μm, while in the graphite-powder-mixed electrolyte, it showed an opposite trend and decreased as the machining depth increased. This was because, in the graphite-powder-mixed electrolyte, high-energy discharge occurring on graphite particles took place at the sidewall of the hole, resulting in material removal from the inner wall of the hole and further causing the small entrance diameter to increase more rapidly than the large entrance diameter. As a result, the HAZ width decreased with increasing machining depth.

### 3.4. Microhole Taper

The taper angle of microholes is an important factor determining the machining accuracy. In order to study the effect of graphite powder on the taper angle of microholes, microholes with a depth of 400 μm were machined under different applied voltages. The small entrance diameter and the bottom diameter of the machined hole were measured using an optical microscope. The hole taper could be calculated using Equation (2), where *θ* represents the taper angle, *D* is the small entrance diameter of the hole, *d* is the bottom diameter of the hole and *h* is the hole depth.

(2) $$\theta =arctan\;(\frac{D-d}{2h})$$

**Figure 8:**
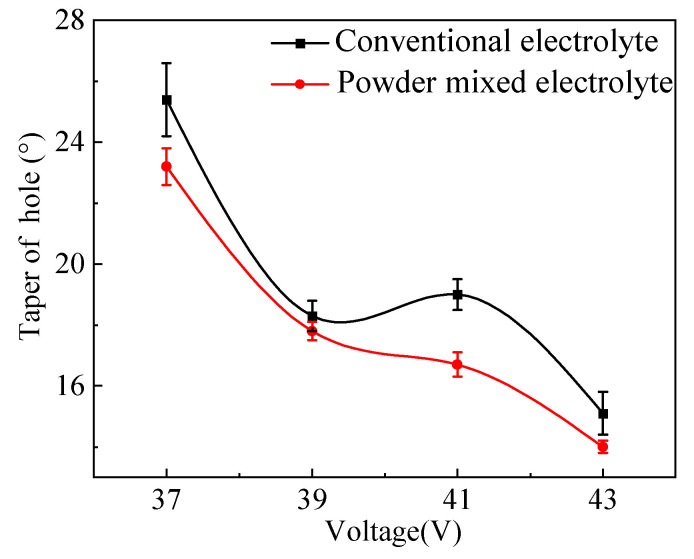
Variation of the hole taper with machining voltage in different electrolytes.

Figure 8 shows the variation of the hole taper angle with increasing machining voltage. It can be seen that for both the conventional electrolyte and the graphite-powder mixed-electrolyte, the hole taper angle decreased with increasing machining voltage. As the machining voltage increased from 37 V to 43 V, the taper angle of the hole obtained in the conventional electrolyte decreased from 25.4° to 15.1°, while for the graphite-powder-mixed electrolyte, the taper angle decreased from 23.3° to 14°. The hole tapers in both electrolytes showed decreasing trends. This was due to the fact that when a low machining voltage was applied, the discharge energy was relatively low, and thus the overcut of the machined hole was relatively small. As the machining depth increased, it became difficult for the electrolyte to flow into the machining zone. The electrolyte and gas bubbles accumulated at the hole entrance. Therefore, more discharges occurred at the entrance, which enlarged the entrance diameter and led to a larger hole taper. When a higher machining voltage was applied, more discharge energy was released, and thus the hole overcut increased accordingly. The electrolyte was relatively easier to flow into the machining zone, which enlarged the bottom diameter of the hole. No significant increase in the hole entrance diameter occurred as the discharge would not concentrate at the hole entrance. Therefore, a smaller hole taper was obtained.

It also can be seen from Figure 8 that the addition of graphite powder into the electrolyte resulted in a decrease in the hole taper angle. The reduction in taper angle was in the range of 0.5° to 2.3° for machining voltage between 37 V and 43 V.

**Figure 9:**
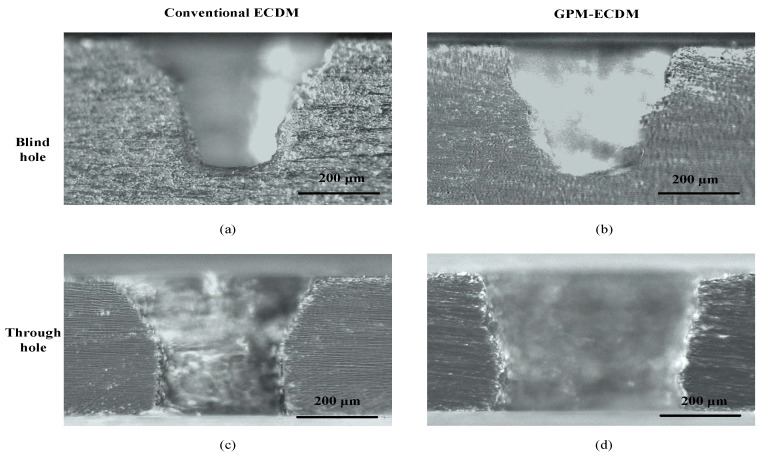
Cross sections of 400 μm depth blind and through holes machined at 37 V. (**a**) Blind hole machined in the conventional electrolyte. (**b**) Blind hole machined in the graphite powder mixed electrolyte. (**c**) Through hole machined in the conventional electrolyte. (**d**) Through hole machined in the graphite powder mixed electrolyte.

Figure 9 shows the cross sections of blind and through holes machined in different electrolytes at 37 V. The depths of both blind and through holes were 400 μm. As shown in Figure 9a,c, both the blind and through holes machined in the conventional electrolyte exhibited inverted cone-shaped cross sections. However, the exit diameter of the through hole was larger than the bottom diameter of the blind hole because, after drilling through the workpiece, the electrode end directly contacted the electrolyte, resulting in significant improvements in the material removal rate around the hole exit. Thus, a larger exit diameter was created. The entrance and exit diameters of the through hole in Figure 9c were measured to be 476 μm and 313 μm, respectively. According to Equation (2), the taper angle of the through hole obtained in the conventional electrolyte was calculated to be 11.5°.

Figure 9b,d show the cross sections of blind and through holes obtained in graphite-powder-mixed electrolyte. Compared with the through hole in Figure 9c, the through hole in Figure 9d had a significant increase in hole diameter. However, the hole sidewall was more vertical. Its taper angle was calculated to be 7.2°. It can be concluded that with the addition of graphite powder into the electrolyte, the taper angle of the through hole decreased from 11.5° to 7.2°, achieving an improvement of 37.4%. However, the hole diameter had increased.

### 3.5. Tool Electrode after Machining

**Figure 10:**
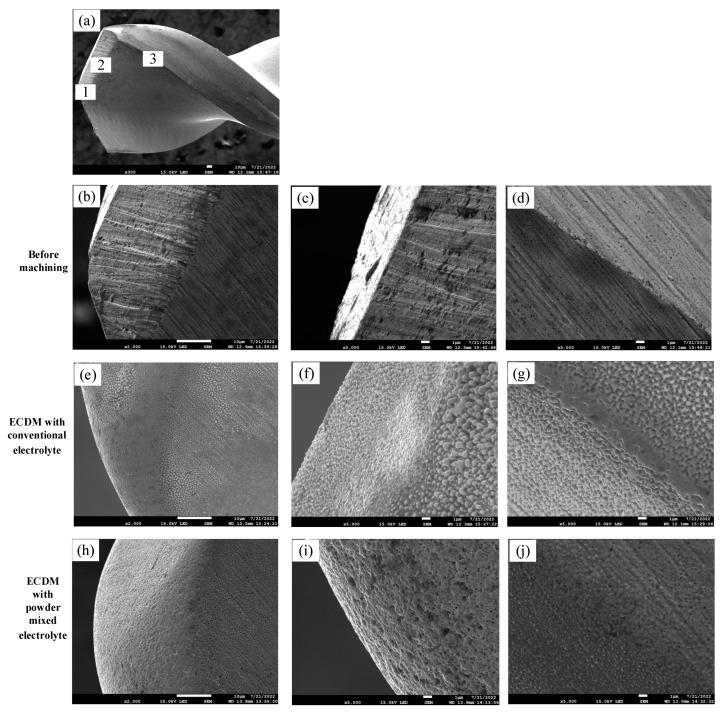
SEM image of tool electrodes. (**a**) Observation regions on the tool electrode: (1) the tip, (2) the cutting edge and (3) the spiral edge. (**b**) The tip, (**c**) the cutting edge and (**d**) the spiral edge on the tool electrode before machining. (**e**) The tip, (**f**) the cutting edge and (**g**) the spiral edge on the tool electrode after ECDM with conventional electrolyte. (**h**) The tip, (**i**) the cutting edge and (**j**) the spiral edge on the tool electrode after ECDM with graphite powder mixed electrolyte.

As the tool electrode used in the experiment has a helical structure, observations were made at the tip, cutting edge and spiral edge of the tool electrode to compare the tool electrode morphology after machining for 20 min. Figure 10 shows the SEM images of the tool electrodes.

As shown in Figure 10, different degrees of wear appeared in the three regions on the tool electrodes. The three regions on the microdrill bit marked with 1, 2 and 3 show visible edges before machining. However, after machining, the edges at the three regions became blunted due to the wear occurring on the surface. Comparing the tool electrodes machined in different electrolytes, it can be seen that the tool electrode surface machined in the conventional electrolyte was uneven and grainy, while the tool electrode surface machined in the graphite-powder-mixed electrolyte showed a honeycomb-like pattern on the spiral edge. This was because the cobalt in the tungsten carbide tool electrode melted and then resolidified on the tool electrode surface in the conventional electrolyte, while in the graphite-powder-mixed electrolyte, the discharge energy was relatively high, and more material on the tool electrode was removed by vaporization. Therefore, the surface morphologies of the tool electrode machined in different electrolytes were different.

**Figure 11:**
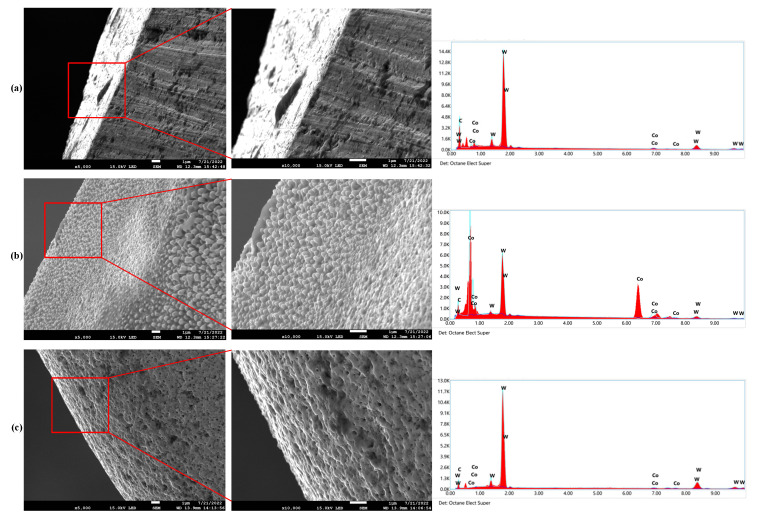
The EDS results of the cutting edge of the tool electrodes before and after machining in two different electrolytes. (**a**) Before machining. (**b**) After machining in conventional electrolyte. (**c**) After machining in graphite-powder-mixed electrolyte.

In order to verify the above analysis, the EDS results of the cutting edge of the tool electrodes before and after machining in two different electrolytes are shown in Figure 11. The tool electrode was a cemented carbide with tungsten carbide as the main component and cobalt as the metal binder. The tungsten carbide has a higher melting point than the cobalt. Therefore, there were three main elements in the tool electrode, which were carbon, tungsten and cobalt.

The EDS spectrum images showed that the cobalt content on the surface of the tool electrode after machining in the conventional electrolyte increased significantly compared to that before machining, while the carbon and tungsten contents decreased. However, the element contents on the surface of the tool electrode machined in the graphite-powder-mixed electrolyte were almost the same as those before machining. The reason for these differences was that for the graphite-powder-mixed electrolyte, as discussed in Section 3.1, the average discharge energy was higher. The cobalt melted and evaporated under a higher discharge energy and was stripped off the surface of the tool electrode as a whole, resulting in a honeycomb-like pattern on the tool electrode surface. However, in the conventional electrolytes, due to the relatively low discharge energy, the cobalt melted due to the discharge and then resolidified on the tool electrode surface after contact with the surrounding electrolyte, resulting in an uneven and granular surface morphology and a significant increase in cobalt content.

## 4. Conclusions

In this study, graphite-powder-mixed electrochemical discharge machining was applied to microhole machining in soda-lime glass. Comparisons of the discharge energy, microhole morphology and tool electrode morphology after machining were made. The following conclusions were obtained.

(1) It was found that there were a lot of small pulse currents distributed on the current waveform when machining with the graphite-powder-mixed electrolyte. These small pulse currents were caused by the discharges between the tool electrode and the graphite powders in the electrolyte. The average discharge energy of the small pulse current was 2.8 times as much as that of the general electrochemical discharge.
(2) Both the large and small entrance diameters of the holes obtained in the graphite-powder-mixed electrolyte were larger than those obtained in the conventional electrolyte when the hole depth was deeper than 200 μm. For the machining voltage of 37–41 V, the HAZ widths in both electrolytes showed increasing trends, and the HAZ width machined in the graphite-powder-mixed electrolyte was larger than that obtained in the conventional electrolyte. However, under the voltage of 43 V, the HAZ width obtained in the graphite-powder-mixed electrolyte decreased with increasing machining depth. In addition, a reduction in hole taper angle with a range of 0.5° to 2.3° was achieved after introducing graphite powder into the electrolyte.
(3) The tool electrode surface machined in the conventional electrolyte was uneven and grainy, which was caused by the molten cobalt resolidifying on the tool electrode surface. However, due to a relatively higher discharge energy in the GPM-ECDM, the tool electrode machined in the graphite-powder-mixed electrolyte showed a honeycomb-like pattern on the surface.

## Figures and Tables

**Table 1:** Material properties of graphite powder.

Parameters	Values
Volume density	1.78–1.82 g/cm^3^
Electrical resistivity	10 μΩ·m
Compression strength	45 MPa
Thermal expansion coefficient	4.8 × 10^−6^/°C

**Table 2:** Microhole machining parameters.

Parameters	Values
Voltage	37 V, 39 V, 41 V, 43 V
Pulse frequency	500 Hz
Duty cycle	50%
Concentration of electrolyte	6 mol/L
Spindle speed	1000 rpm
Feed speed	1 μm/s
Machining depth	100 μm, 200 μm, 300 μm, 400 μm
Graphite powder concentration	0.5 wt %

## Data Availability

Not applicable.
